# Magnitude of urban household food insecurity in East Africa: a systematic review and meta-analysis

**DOI:** 10.1017/S1368980021003529

**Published:** 2022-04

**Authors:** Bereket Gebremichael, Biruk Beletew, Melaku Bimerew, Demewoz Haile, Sibhatu Biadgilign, Kaleab Baye

**Affiliations:** 1 College of Health Science, Addis Ababa University, PO Box 13386, Addis Ababa, Ethiopia; 2 Woldia University, College of Health Sciences, Department of Nursing, Woldia, Ethiopia; 3 Department of Health Studies, College of Human Science, University of South Africa, Pretoria, UNISA, South Africa; 4 Center of Food Science and Nutrition, Addis Ababa University, Addis Ababa, Ethiopia

**Keywords:** Urban household, Food insecurity, Eastern Africa, Meta-analysis

## Abstract

**Objective:**

The purpose of this study was to determine the magnitude and determinants of urban household food insecurity in East Africa.

**Design:**

Systematic review and meta-analysis.

**Setting:**

Studies conducted in East Africa.

**Participants:**

Seventeen studies (fifteen cross-sectional and two cohort) that enrolled 156 996 households. We used the Preferred Reporting Items for Systematic Review and Meta-Analysis (PRISMA) guidelines to search electronic databases (PubMed, Cochrane Library, EMBASE, CINAHL, African Journals OnLine, Web of Science, Scopus and Google Scholar; date of last search: 10 June 2020) for studies reporting the prevalence and associated factors of urban household food insecurity.

**Results:**

A total of 17 studies with 156 996 households from 8 countries were used for the analysis. The pooled prevalence of urban household food insecurity in East Africa was 60·91 % (95 % CI 47·72, 74·11; *I* ^2^ = 100 %; *P* < 0·001) where the highest (91 %) and lowest (36·5 %) was observed in Sudan and Burundi, respectively. Household head educational status (illiterate) (AOR = 2·53; 95 % CI 2·11, 2·95, *I* ^2^ = 90 %; *P* < 0·01), female as household head (AOR = 1·45; 95 % CI 1·16, 1·75; *I* ^2^ = 0·0 %; *P* = 0·993), large family size (AOR = 1·43; 95 % CI 1·09, 1·76, *I* ^2^ = 0·0 %; *P* = 0·863) and poorest wealth quantile (AOR = 3·95; 95 % CI 1·93, 5·98; *I* ^2^ = 57·2 %, *P* = 0·053) were factors which significantly increased odds of urban household food insecurity in East Africa.

**Conclusions:**

The prevalence of urban household food insecurity in East Africa remains high. Therefore, policies and intervention programmes should be designed to reduce the high burden of food insecurity among urban households considering the identified factors.

Food and nutrition insecurity are serious public health challenges affecting millions of households globally^(1)^. The United Nations (UN) agencies reported in 2019 that more than 2 billion individuals do not have daily access to healthy, nutritious and adequate food, and one in nine individuals faces chronic food insufficiency^(2,3)^. Africa has the highest number of undernourishment, with food insecurity being one in every four citizens^(4)^.

Food insecurity is becoming a major threat for contemporary societies with both short- and long-term impacts on human survival and well-being^(5)^. It has been linked to a variety of health outcomes such as under nutrition^(6–9)^, iron deficiency anaemia, multiple chronic conditions (diabetes, kidney disease and CVD), obesity^(10–12)^, mental illness (depression)^(13)^ and risky sexual behaviours (HIV/AIDS)^(7,14,15)^. There is an increasing evidence representing that in young and school-age children, food insecurity is associated with the risk of developmental problems, which affects their school performances^(16)^. Furthermore, food insecurity has a great impact on healthcare burden and healthcare costs^(17)^.

Approximately 55 % of the global population currently resides in urban areas and this number is expected to increase to 68 % by 2050 due to rural–urban migration^(18)^. Inequalities, in terms of infrastructure, services, social amenities and economic activities in favour of urban centres resulted in rise of urban poor due to rural–urban migration^(19,20)^. Many rural migrants are less educated and unskilled and end up in low-income, informal sectors^(21)^. As a result of rapid urbanisation with unparalleled economic growth, urban growth in developing countries is followed by swift expansion of unplanned, disadvantaged neighbourhoods/slums with large concentration of poor urban population hence leading to high burden of urban food insecurity^(5,22)^.

A number of studies have been conducted to assess the magnitude and determinants of urban household food insecurity in East African countries^(23)^. However, results were inconsistent with a reported magnitude ranging from 20·5 % to 93 %^(24)^. Rigorous evaluations of the magnitude and factors associated with urban food insecurity are needed to inform interventions that support the Sustainable Development Goals which aims zero hunger by 2030. This systematic review and meta-analysis were therefore carried out to determine the magnitude and determinant of urban household food insecurity in East African countries.

## Methods

### Reporting

We used the Preferred Reporting Items for Systematic Review and Meta-Analysis statement (PRISMA) guideline to report the finding^(25)^. The article screening and selection process was depicted using a PRISMA flow diagram^(26)^.

### Searching strategy and information sources

We reported studies providing data from PubMed, Cochrane library, EMBASE, CINAHL, African Journals OnLine, Web of Science and Scopus and Google Scholar on the prevalence and possible risk factors of household food insecurity in urban residents, with the search focusing on Eastern Africa. To find additional appropriate studies for this study, we also screened the reference lists of the remaining articles. We contacted the corresponding authors to handle articles with incomplete recorded data. Additional grey literatures were also collected from foreign and local organisations and universities’ official websites. The search included MeSH terms and keywords independently and/or in combination using Boolean operators ‘OR’ or ‘AND’. The core search terms and phrases included ‘prevalence’, ‘magnitude’, ‘burden’, ‘causes’, ‘determinants’, ‘associated factors’, ‘predictors’ ‘risk factors’, ‘urban’, ‘city’, ‘town’, ‘municipal’, ‘metropolitan’, ‘household’, ‘domestic’, ‘family’, ‘food insecurity’, ‘food security’ and ‘East Africa’. In PubMed database, the following search strategy was used: (prevalence OR magnitude OR epidemiology) AND (causes OR determinants OR associated factors OR predictors OR risk factors) AND (urban (MeSH Terms) OR city OR town OR municipal OR metropolitan) AND (household (MeSH Terms) OR domestic OR family) AND (food insecurity (MeSH Terms) OR food security) AND Eastern Africa.

### Study selection and data extraction

Retrieved studies were exported to Endnote software, version 8, and duplicate studies were removed. The authors created a data extraction form that includes the name of the author, year of publication, country of study, study design, sample size, prevalence of urban household food insecurity and identified categories of factors associated with food insecurity. The data abstraction form was piloted using four randomly chosen papers from the data base. After having piloted the template, the extraction form was amended. Before full-text articles retrieval, two authors (BB and MB) independently screened the selected studies using their titles and abstracts. To further screen the full-text articles, we used pre-specified inclusion criteria. Differences were addressed and resolved through a consensus meeting with other reviewer (BG), for the final selection of studies to be included in the systematic review and meta-analysis.

### Inclusion and exclusion criteria

We included observational studies (cross-sectional and cohort studies). Studies published in English between 2005 and 2020, reporting the prevalence and/or at least one associated factor with urban household food insecurity in Eastern Africa countries, were considered. With the goal of providing a valid instrument for use in developing countries, Food and Nutrition Technical Assistance Project (FANTA) developed the Household Food Insecurity Access Scale (HFIAS) between 2001 and 2006. Hence, the HFIAS was globally used to quantify food insecurity during this period^(27)^ which is why we limited our review to start from 2005. Unpublished (grey literature) from credible sources were also considered.

### Quality assessment

For quality assessment of the published reports, the Joanna Briggs Institute (JBI) quality assessment checklist was used^(28,29)^. Cross-sectional and cohort studies with scores of 5 and above were considered as low risk or good quality^(28,29)^, while those with scores of 4 and below were considered as high risk or poor quality and were therefore omitted (see online Supplemental Table 1).

### Outcome measurement

The main outcome was urban household food insecurity. All the seventeen studies in the meta-analysis used HFIAS to determine household food insecurity status.

### Statistical analysis

We imported the data to STATA version 14.0 statistical software for further analysis after the data were extracted using the Microsoft Excel format. Standard errors for each sample were determined using the binomial distribution formula. Pooled estimates of the magnitude of urban household food insecurity was estimated by a random effect model^(30)^. We used forest plot to show the pooled prevalence with 95 % CI of urban household food insecurity. OR with 95 % CI was also presented in forest plot to show factors associated with urban household food insecurity. Cochrane’s Q statistics (*χ* ^2^), *I* ^2^ (inverse variance weighting) and *P*-values were used to examine the heterogeneity^(31)^. The *I* ^2^ statistical value of 0 in this analysis shows homogeneity, while the values of 25, 50 and 75 percent showed medium, moderate and strong heterogeneity, respectively^(32,33)^. Owing to a small number of studies, variability in the study design and settings in our analysis which would likely introduce heterogeneity, random effects model was used for the analysis.

For variables that showed high heterogeneity, we used narrative synthesis to summarise the finding in addition to reporting the pooled estimate. We used sensitivity analysis by removing outlier study to see the effect of a single study on the overall estimation. Funnel plot and Egger’s regression test were used to check publication bias. Sensitivity analysis was done for those studies that showed potential publication bias by removing them^(34)^.

## Result

### Study selection

**Fig. 1:**
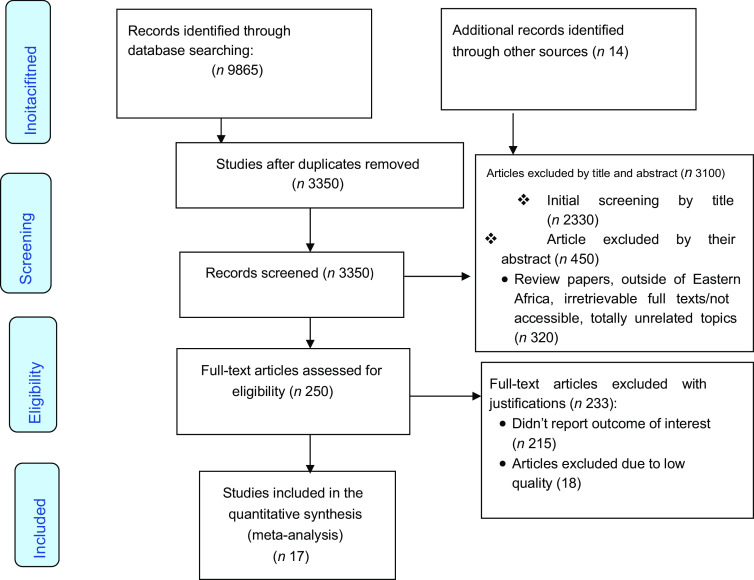
PRISMA flow diagram showing the results of the search and reasons for exclusion. PRISMA, Preferred Reporting Items for Systematic Review and Meta-Analysis

Overall, 9879 studies that were conducted between 2005 and 2020 were identified by electronic searches (9865 studies via database searching and 14 studies from other sources). After excluding duplicates, a total of 3350 papers remained. Finally, 250 studies were screened for full-text review and, 17^(35–51)^ articles (*n* 156, 996 households) were selected for the final analysis (Fig. 1).

### Characteristics of included studies

The characteristics of the seventeen studies included in the systematic review and meta-analysis are summarised in Table 1 ^(35–51)^. Regarding the countries where the studies were conducted, six studies were done in Ethiopia^(35–40)^, four in Kenya^(41–43,46)^, one in Mozambique^(50)^, one in Burundi^(44)^, two in Rwanda^(47,48)^, one in Somalia^(49)^, one in Sudan^(45)^ and one in Uganda^(51)^. The studies included households, ranging from 103^(50)^ to 84 756^(48)^ (Table 1).

**Table 1:** Descriptive summary of seventeen studies included in the systematic review and meta-analysis on the prevalence and determinants of household food insecurity in East Africa, 2005–2020

Author, reference	Year	Country	Study design	Sample size	Prevalence (%)	Quality score
1. Feleke *et al*.^(35)^	2009	Ethiopia	Cross-sectional	200	43	6/8
2. Gebre *et al*.^(36)^	2012	Ethiopia	Cross-sectional	423	58·16	7/8
3. Birhane *et al*.^(37)^	2014	Ethiopia	Cross-sectional	550	75	7/8
4. Tefera *et al*.^(38)^	2011	Ethiopia	Cross-sectional	140	48	7/8
5. Tantu *et al.* ^(39)^	2017	Ethiopia	Cross-sectional	609	37·6	8/10
6. Etana *et al.* ^(40)^	2017	Ethiopia	Cross-sectional	410	87·6	7/10
7. Kimani-Murage *et al.* ^(41)^	2014	Kenya	Cohort	3000	85	6/8
8. Mutisya *et al.* ^(42)^	2016	Kenya	Cohort	56 344	41·86	5/8
9. Webb-Girard *et al.* ^(43)^	2012	Kenya	Cross-sectional	150	77	8/10
10. Mairie *et al.* ^(44)^	2008	Burndi	Cross-sectional	200	36·5	8/8
11. Bushara *et al*.^(45)^	2016	Sudan	Cross-sectional	394	91	7/8
12. Owuors *et al.* ^(46)^	2018	Kenya	Cross-sectional	1434	62	6/8
13. Foeken *et al*.^(47)^	2015	Rwanda	Cross-sectional	7500	21	7/8
14. Gill *et al.* ^(48)^	2016	Rwanda	Cross-sectional	84 756	80·6	7/8
15. Oloo *et al.* ^(49)^	2014	Somalia	Cross-sectional	246	37	8/8
16. Ganhão *et al.* ^(50)^	2015	Mozambique	Cross-sectional	103	65	7/8
17. Nantale *et al*.^(51)^	2017	Uganda	Cross-sectional	537	88·5	7/8

### Meta-analysis

#### Prevalence of urban household food insecurity in East Africa

**Fig. 2:**
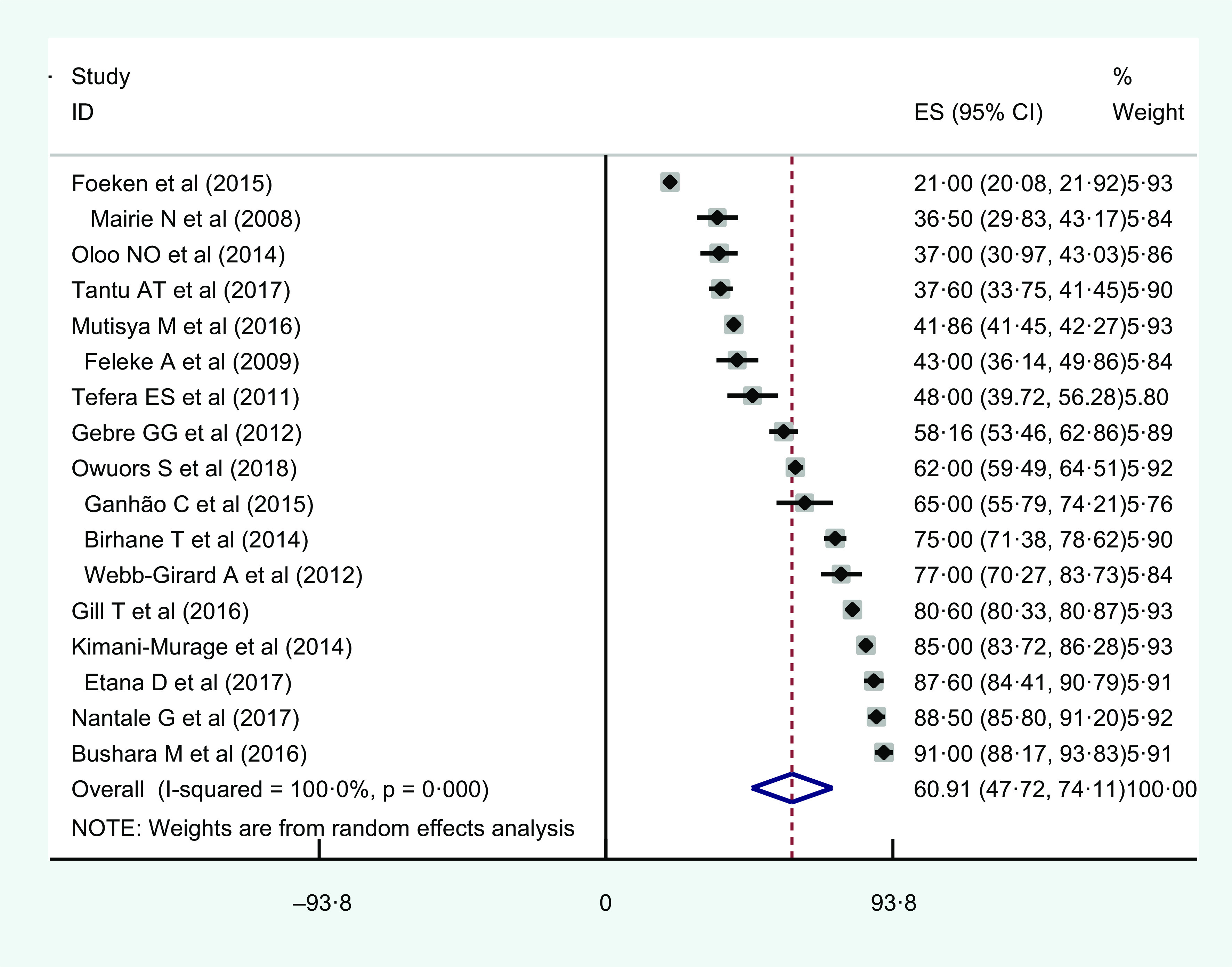
Forest plot of the pooled prevalence of urban household food insecurity in Eastern Africa, 2005–2020

The prevalence of household food insecurity was reported by all of the studies (*n* 17)^(35–51)^. The prevalence of urban household food insecurity ranged from 21 % in Kenya^(47)^ up to 91 % in Sudan^(45)^. Based on the random effects model analysis, the pooled prevalence of urban household food insecurity in East Africa was estimated to be 60·91 % (95 % CI 47·72, 74·11; *I* ^2^ = 100 %; *P* < 0·001) (Fig. 2).

#### Sensitivity analysis

The results of the sensitivity analysis showed that our findings were not dependent on a single study. The 95 % CI of the pooled prevalence of urban household food insecurity overlapped after removing a single study in the sensitivity analysis but varied between 58·3 % (95 % CI 45·82, 70·72)^(45)^ and 70·0 % (95 % CI 50·46, 73·53)^(47)^ (see online Supplemental Fig. 1).

#### Publication bias

We checked for possible publication bias and a funnel plot demonstrated symmetrical distribution. The *P*-value of the Egger regression test was 0·475, which showed the low publication bias risk (see online Supplemental Fig. 2).

#### Factors associated with urban household food insecurity

Nine studies out of the total seventeen studies included in this meta-analysis reported on factors associated with urban food insecurity (Table 2).

**Table 2:** Meta-regression included studies on factors associated with household food insecurity in East Africa

Variables	OR	95 % CI	Author (reference)	Pooled AOR	95 %CI	*I* ^2^	*P*-value
Educational status (illiterate)	1·33	0·76, 2·32	Tantu *et al.*	2·53	2·11, 2·95	90 %	<0·01
	6·03	2·2, 11·4	Feleke *et al.*				
	3·4	1·7, 6·7	Birhane *et al.*				
	3·66	1·4, 8·8	Etana *et al.*				
	2·7	0·58, 13·25	Nantale *et al.*				
Female household head	1·3	0·39, 4·28	Tantu *et al.*	1·45	1·16, 1·75	0·0 %	0·9937
	1·5	1·1, 1·9	Feleke *et al.*				
	1·4	1·01, 1·91	Etana *et al*.				
Family size	1·51	1·2, 2·71	Foeken *et al.*	1·43	1·09, 1·76	0·0 %	0·863
	1·25	0·76, 2·32	Tantu *et al.*				
	1·4	1·01, 1·91	Mutisya *et al.*				
	1·9	1·02, 3·68	Nantale *et al.*				
Wealth index	3·8	1·5, 9·7	Mairie *et al.*	3·95	1·93, 5·98	57·2 %	0·053
	9·5	2·1, 12·6	Tantu *et al.*				
	6·7	2·2, 11·4	Mutisya *et al.*				
	2·64	1·02, 4·21	Feleke *et al.*				
	2·20	0·30, 4·10	Bushara *et al.*				

References: Male household head, Small family size (≤ 4), Richest wealth quantile, Literate (Attended higher education or above).

#### Female-headed households

Three studies found a significant association between female-headed households and urban household food insecurity. Of these, the highest and lowest AOR (95 % CI) was 1·5 (95 % CI 1·1, 1·91)^(35)^ and 1·3 (95 % CI 0·39, 4·28)^(39)^, respectively (Table 2).

**Fig. 3:**
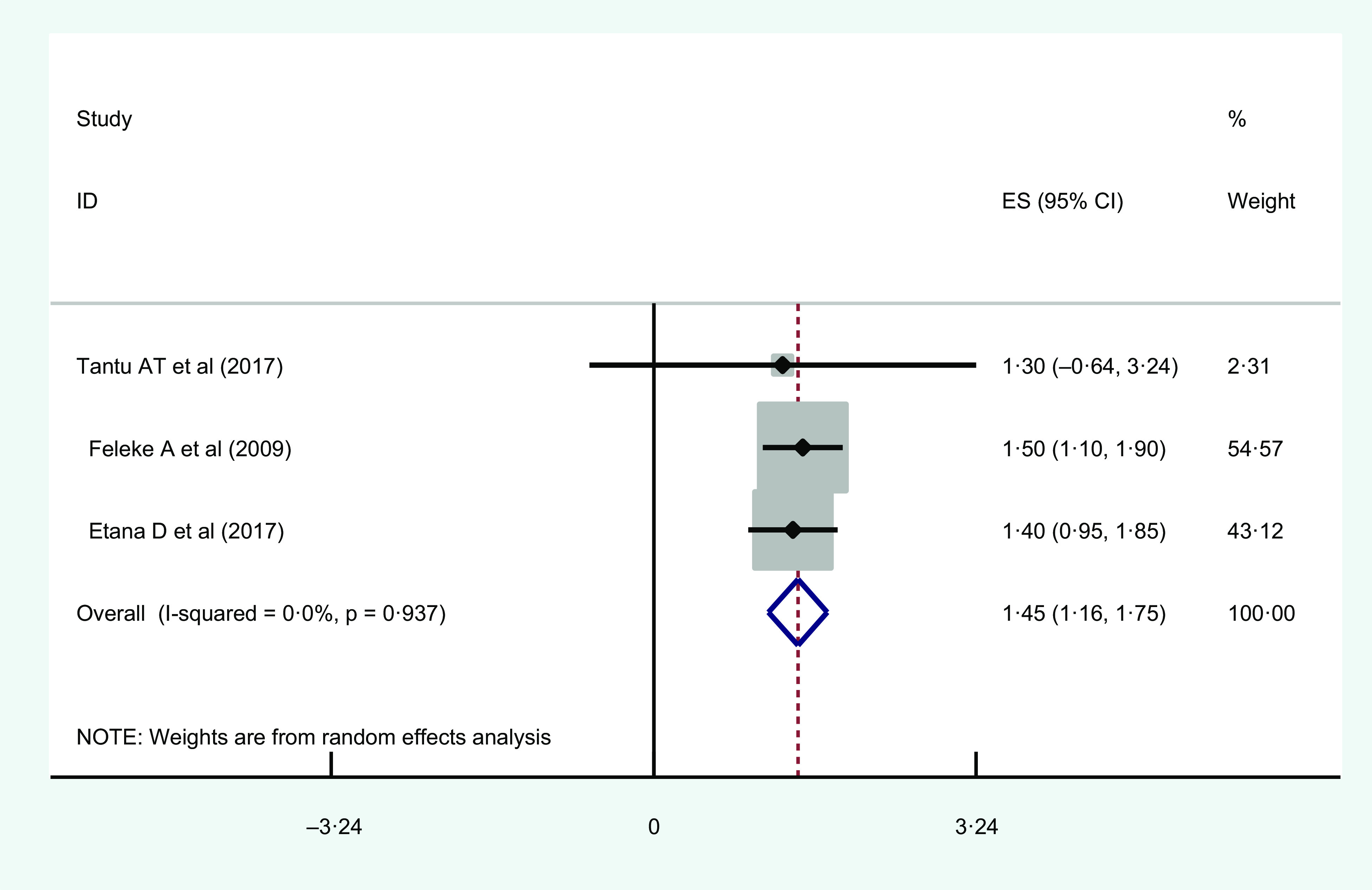
Forest plot showing the pooled estimates of female-headed household as a risk factor for urban household food insecurity in Eastern Africa

Regarding the heterogeneity test, Galbraith plot showed the heterogeneity is very low. The combined result based on the two studies showed an overall estimate AOR of female-headed household as a determinant of urban household food insecurity was 1·45 (95 % CI 1·16, 1·75; *I* ^2^ = 0·0 %; *P* = 0·9937) (Fig. 3).

A funnel plot revealed a symmetrical distribution with respect to the test for publication bias. Egger’s regression test *P*-value was 0·649, which revealed the absence of publication bias (see online Supplemental Fig. 3).

Sensitivity analysis was used to check if there is possible source of heterogeneity in the pooled estimate of female as a household head as a risk factor of urban household food insecurity. The finding showed that our result was not dependent on a single study (see online Supplemental Fig. 4).

#### Educational status

Five of the studies reported that illiteracy (no formal education) of head of the household was significantly associated with urban food insecurity compared to household headed by those who attended higher education. Among this, the highest AOR = 6·03 (95 % CI 2·2, 11·4)^(40)^ and the lowest AOR = 1·33 (95 % CI 0·76, 2·32)^(39)^ were observed among those who were illiterate (no formal education) when compared with those educated (Table 2).

**Fig. 4:**
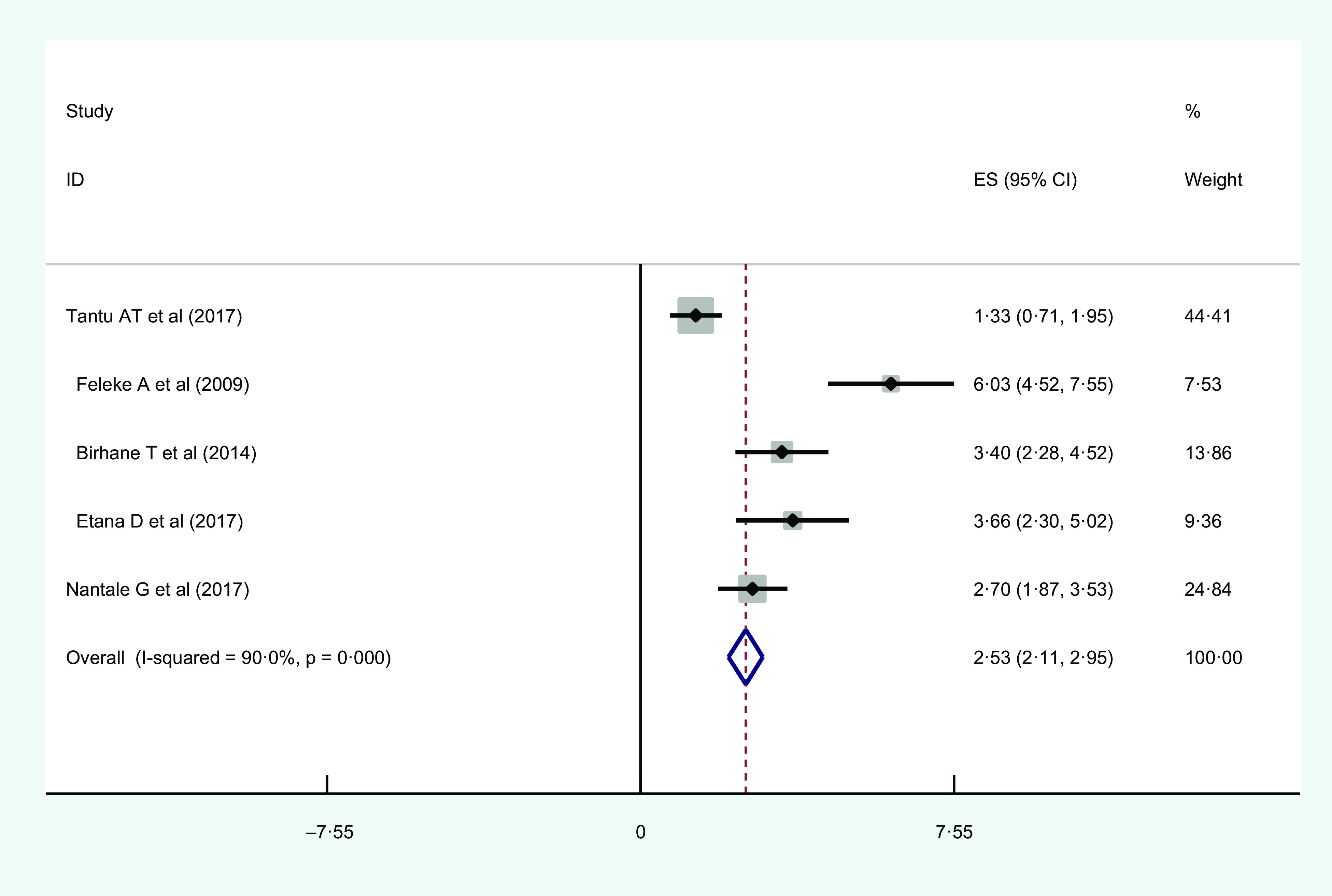
Forest plot showing the pooled estimates of illiteracy as risk factors of urban household food insecurity in Eastern Africa

The overall estimated AOR of illiteracy as a risk factor for urban household food insecurity was 2·53 (95 % CI 2·11, 2·95; *I* ^2^ = 90 %; *P* = < 0·01). Regarding the heterogeneity, *I*-squared (*I* ^2^) and *P*-value showed heterogeneity. Galbraith plot also showed heterogeneity. Therefore, it should be noted that the interpretation of the pooled estimate should be made with caution as there was heterogeneity (Fig. 4).

Regarding publication bias, both the funnel plot and Egger’s regression test *P*-value indicated the absence of publication bias (see online Supplemental Fig. 5).

The results of the sensitivity analysis showed that our findings were not dependent on a single study (see online Supplemental Fig. 6).

#### Family size

Large family size was considered when number of family is greater than or equal to 5. Four studies found a significant association between increased family size and urban household food insecurity. The highest AOR = 1·9 (95 % CI 1·02, 3·68) was observed in Sudan^(45)^ and lowest AOR = 1·25 (95 % CI 0·76, 2·32) was observed in Ethiopia^(39)^ (Table 2).

**Fig. 5:**
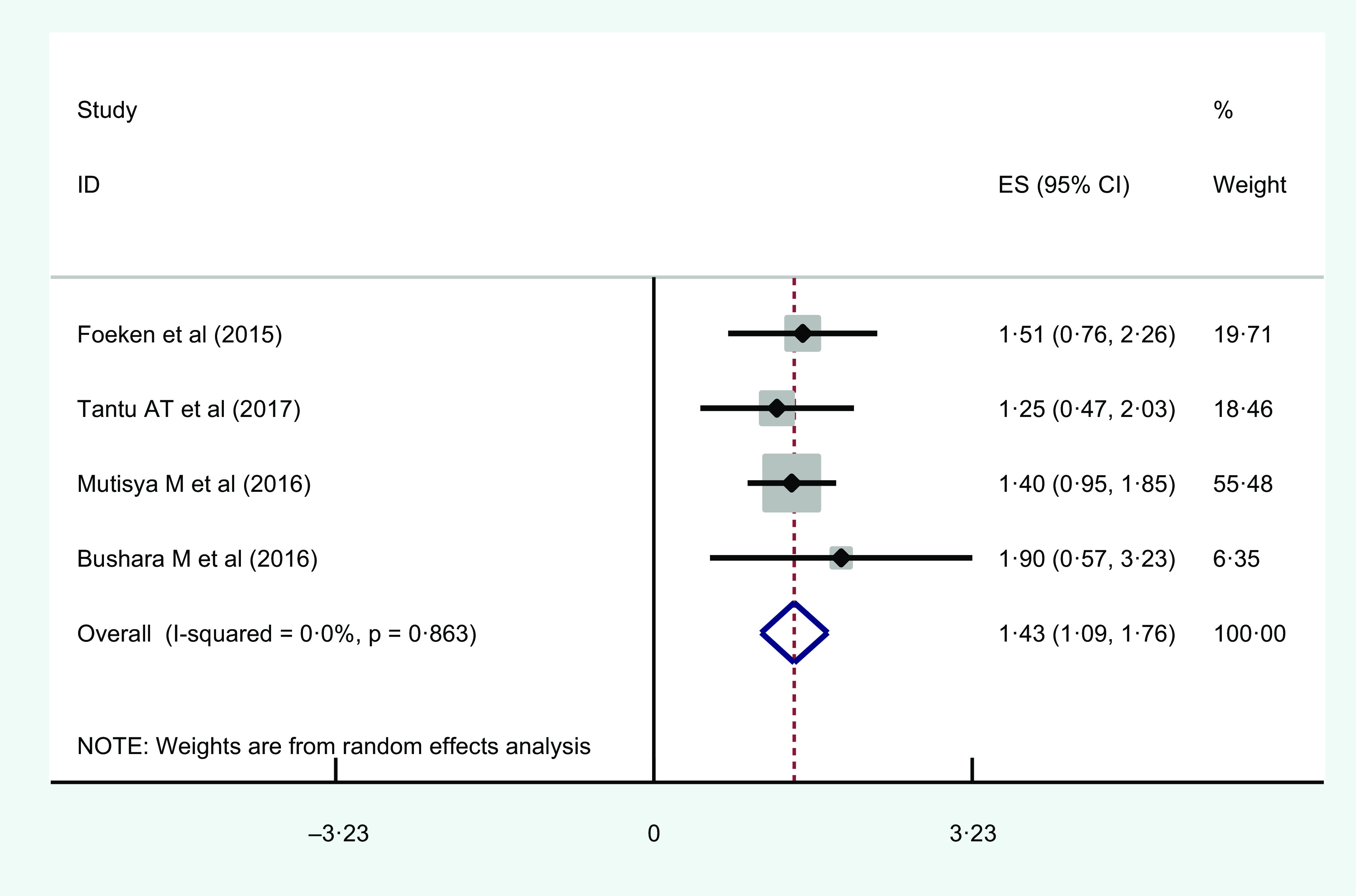
Forest plot showing the pooled estimate of increased family size as risk factors of urban household food insecurity in Eastern Africa

The meta regression showed that having large family size increase odds of food insecurity by 43 %, that is, 1·43 (95 % CI 1·09, 1·76; *I* ^2^ = 0·0 %; *P* = 0·863) (Fig. 5).

Regarding the test of publication bias, both the funnel plot and Egger’s regression test *P*-value indicated the absence of publication bias (see online Supplemental Fig. 7). Based on the leave-one-out sensitivity analysis, our findings were not dependent on a single study (see online Supplemental Fig. 8).

#### Wealth index

Five studies found a significant association between lowest wealth quantile and urban household food insecurity. The highest AOR = 9·5 (95 % CI 2·1, 12·6) was observed in Ethiopia^(39)^ and lowest AOR = 2·2 (95 % CI 1·05, 4·86) was observed in Sudan^(45)^ (Table 2).

**Fig. 6:**
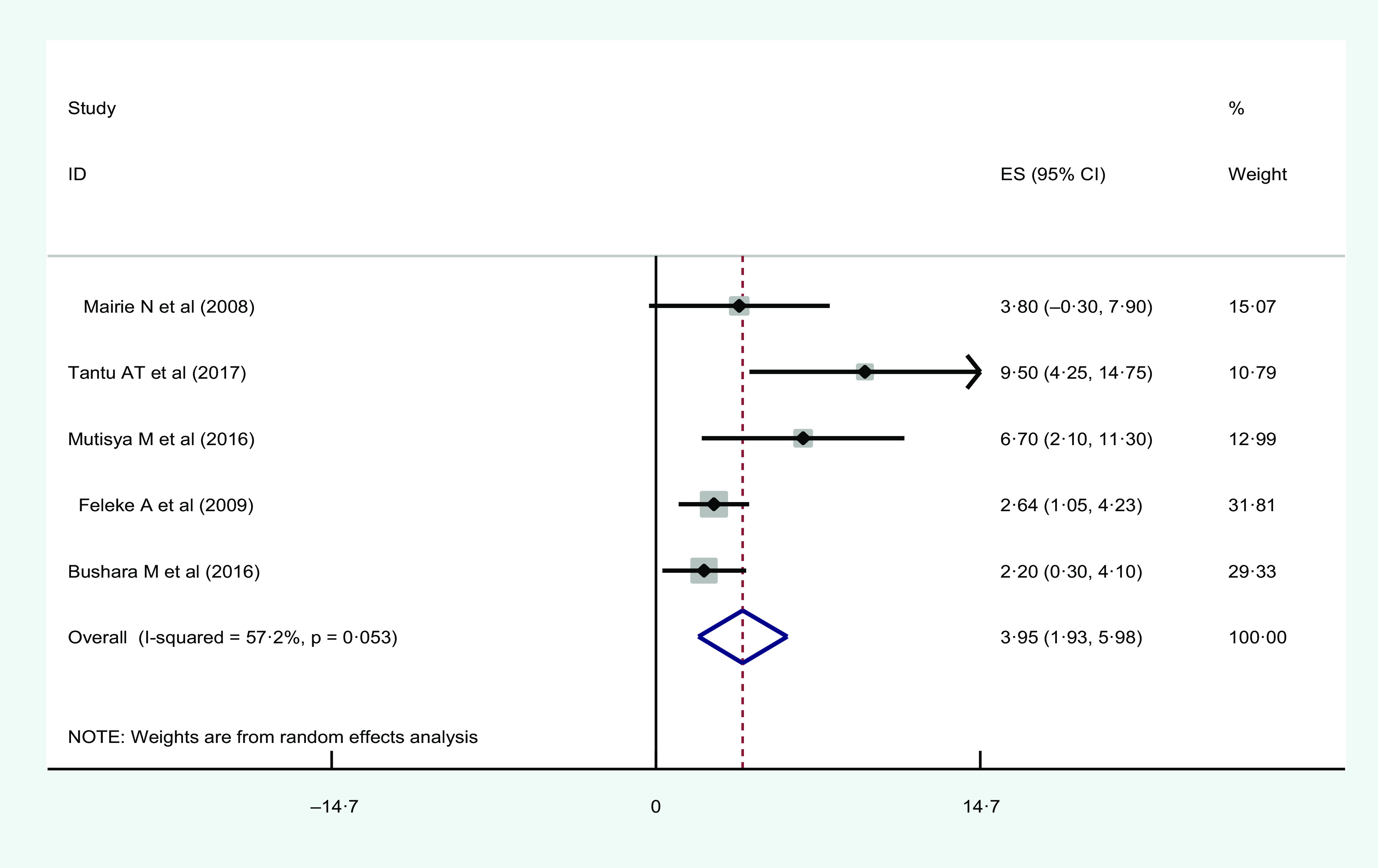
Forest plot showing the pooled estimate of lowest wealth quantile as risk factors of urban household food insecurity in Eastern Africa

The forest plot showed the overall estimated AOR of lowest wealth quantile as a determinant factor for urban household food insecurity was 3·95 (95 % CI 1·93, 5·98; *I* ^2^ = 57·2 %; *P* = 0·053) (Fig. 6).

Publication bias was checked using a funnel plot. The finding showed that there was asymmetrical distribution. Egger’s regression test *P*-value (0·045) also indicated the presence of publication bias (see online Supplemental Fig. 9). Therefore, we performed a trim and fill analysis. After the trim and fill analysis, the overall estimated AOR of low family income as a determinant of urban household food insecurity became 3·95 (95 % CI 1·93, 5·98) (see online Supplemental Fig. 10).

Moreover, the results of the sensitivity analysis showed that our findings were not dependent on a single study (see online Supplemental Fig. 11).

## Discussion

These systematic review and meta-analysis were carried out to determine the magnitude and factors associated with urban household food insecurity in East Africa. In the final analysis, seventeen studies were included and the combined magnitude of urban household food insecurity was found to be high in East Africa. Factors like being uneducated, household headed by female, increased family size and low household income were found to be associated with urban household food insecurity in the region.

Urbanisation poses a big challenge to food availability in terms of changing consumption patterns and supply processes^(5)^. Due to the combination of high urban poverty rates, high reliance of urban households on market-supplied food and fluctuating food prices, urban food insecurity is a rising concern^(37)^. Similarly, the result of this systematic review and meta-analysis showed that more than half (60·91 %) of urban households in East Africa were food-insecure. The finding was comparable with a study conducted in South Africa and Brazil^(52,53)^. However, the finding of this study was lower than results reported from the eleven cities in southern Africa and Southwest Nigeria^(54,55)^. This discrepancy might be attributed to methodological differences (differences in operational definition and measurement scale for food insecurity). On the other hand, our findings reported higher levels than those reported from Iran^(55)^, Canada^(56)^ and USA^(57)^. The possible explanation could be socio-economic differences, as countries like Canada, USA, Iran and are more developed than countries in East Africa.

This study revealed several factors associated with household food insecurity. Higher odds of food insecurity were observed in households headed by female compared to their counterparts. Similar findings were observed in other studies^(53,58,59)^. This could be due to the deep-rooted gender inequalities which affect females across the world. Mainly, women in developing countries are affected by inequalities and discrimination which results in low rate of education and employment^(60)^. Women tend to be majorly involved in childbearing and home activities which could be a barrier for career development to get good paying jobs in the highly competitive urban job opportunity. Due to this reason, they will not have enough time for paid job. Furthermore, in most countries of the world, resources like lands are owned by males^(61)^. Therefore, females are vulnerable to low-income jobs, which lead to food insecurity^(62–64)^.

Lower levels of education, especially illiteracy, was also found to increase the odds of food insecurity among urban households in East Africa. This result was in line with a systematic review conducted from studies conducted in sub-Saharan countries^(59)^. Similarly, studies conducted in Nigeria, South Africa^(52)^, North India^(65)^, Iran^(66)^ and USA^(67)^ also found association between low educational attainment and food insecurity. The possible justification could be lower levels of education leads to lower skills which end up with unemployment/employment in informal sectors. This in turn leads to low payment and lower purchasing abilities. Lower levels of education also lead to low ability in managing and planning food expenditures and food utilisation, which in turn lead to food insecurity.

Urban households mainly depend on purchasing to access their daily food. Households with lower incomes cannot afford the rising cost of food specially in urban areas which results in food insecurity^(68,69)^. Our study also found that households with low income were found to have higher odds of food insecurity than their counterparts. This finding was in line with studies conducted in sub-Saharan Africa^(59)^, South and Southern Africa^(52,54)^, Nigeria, Brazil^(53)^, North India^(65)^ and USA^(67)^. In the contrary to this, food insecurity is also recorded in high-income households. Various reasons like unusually high economic needs, difference in intrahousehold allocation of money and change in household composition have been postulated to justify that. There are also non-economic causes of food insecurity^(70)^.

Family size was another factor that was significantly associated with urban food insecurity in this study. Households with higher number of family members were found to have higher odds of food insecurity than households with fewer family members. This was consistent with findings conducted in sub-Saharan Africa^(59)^, Nigeria, Iran^(66)^ and India^(71)^. The likely explanation behind the association between family size and food insecurity might be due to the fact that households with increased family size will have increased demand of food and other living expenses which jeopardise food security status of the household.

Various interventions are being implemented to combat food insecurity in the region. But the interventions mainly focus on rural community^(72,73)^. As food security is highly wide spreading in urban areas, interventions that focuses only on rural area alone are unlikely to bring about impactful change. Although few interventions are implemented in urban setups, they mainly focus on general and specific economic interventions (e.g. cash transfer programme) which only improves a single aspect of food security (access to food)^(73,74)^. Based on our study, various factors were found to be associated with household food insecurity in the region. To enhance all aspects of food security, the already established intervention should integrate other interventions that also targets women empowerment, family planning and education sectors.

### Strengths and limitations

This study has several strengths: we used a pre-specified search strategy and data abstraction protocol. In addition, we used internationally accepted tools for a critical appraisal of individual studies. Besides, we performed publication bias and sensitivity analysis. Nevertheless, this review had some limitations: because of the inclusion of studies which are published in English only, language bias is likely. In addition, included studies which reported the outcome of interest were not from all East African countries which affects its representativeness. Furthermore, due to limited number of studies included, high heterogeneity was observed for some of the variable of interest. Therefore, caution should be taken in the interpretation of the result.

## Conclusion and recommendations

The magnitude of urban household food insecurity found to be high in East Africa. Family size, educational status, lower income and being a female-headed household were identified factors that have significant association with urban household food insecurity. Because of the study design, we cannot determine whether the observed associations between food insecurity and the identified factors are causal in nature. However, these observations are valuable in identifying subgroups of the population who are at highest risk for food insecurity and should be targeted for interventions. Therefore, comprehensive policies and intervention programmes should be designed to reduce the high burden of food insecurity among urban residents considering the identified factors.

## Supplementary material

For supplementary material accompanying this paper visit https://doi.org/10.1017/S1368980021003529. click here to view supplementary material
