# Supplementary material for: Magnitude of urban household food insecurity in East Africa: a systematic review and meta-analysis
Source: Public Health Nutr. 2021 Aug 16;25(4):994–1004. doi: 10.1017/S1368980021003529 (PMC9991803; doi:10.1017/S1368980021003529)
Supplement: Supplementary file 1 [file S1368980021003529sup.zip › S1368980021003529sup001.docx]

Supplementary Table 1: Quality appraisal result of included studies in East Africa using Joanna Briggs Institute (JBI) quality appraisal checklist

| **Author** | **Quality assessment questions** | | | | | | | | | | |  |  |  |
| --- | --- | --- | --- | --- | --- | --- | --- | --- | --- | --- | --- | --- | --- | --- |
|  | Q1 | Q2 | Q3 | Q4 | Q5 | Q6 | Q7 | Q8 | Q9 | Q10 | Q11 | Yes Total | Quality status | Overall appraisal |
| Cross-sectional studies | | | | | | | | | | | | | | |
| 1. **Feleke A *et al*** | Y | Y | N | Y | Y | N | Y | Y |  |  |  | 6/8 | Low risk | Included |
| 1. **Gebre GG *et al*** | Y | Y | UC | Y | Y | Y | Y | Y |  |  |  | 7/8 | Low risk | Included |
| 1. **Birhane T *et al*** | Y | Y | Y | Y | Y | N | Y | Y |  |  |  | 7/8 | Low risk | Included |
| 1. **Tefera ES *et al*** | Y | Y | Y | Y | Y | UC | Y | Y |  |  |  | 7/8 | Low risk | Included |
| 1. **Tantu AT *et al*** | Y | Y | Y | Y | Y | Y | Y | Y |  |  |  | 8/8 | Low risk | Included |
| 1. **Etana D *et al*** | Y | Y | Y | Y | Y | UC | Y | Y |  |  |  | 7/8 | Low risk | Included |
| 1. **Kimani-Murage *et al*** | Y | N | Y | Y | Y | UC | Y | Y |  |  |  | 6/8 | Low risk | Included |
| 1. **Mutisya M *et al*** | Y | UC | Y | Y | Y | N | Y | Y |  |  |  | 6/8 | Low risk | Included |
| 1. **Webb-Girard A *et al*** | Y | Y | Y | Y | Y | Y | Y | Y |  |  |  | 8/8 | Low risk | Included |
| 1. **Mairie N *et al*** | Y | Y | Y | Y | Y | Y | Y | Y |  |  |  | 8/8 | Low risk | Included |
| 1. **Bushara M *et al*** | UC | Y | Y | Y | Y | Y | Y | Y |  |  |  | 7/8 | Low risk | Included |
| 1. **Owuors S et al** | Y | Y | UC | Y | Y | N | Y | Y |  |  |  | 6/8 | Low risk | Included |
| 1. **Foeken *et al*** | Y | Y | Y | Y | Y | UC | Y | Y |  |  |  | 7/8 | Low risk | Included |
| 1. **Gill T *et al*** | UC | Y | Y | Y | Y | Y | Y | Y |  |  |  | 7/8 | Low risk | Included |
| 1. **Oloo NO *et al*** | Y | Y | Y | Y | Y | Y | Y | Y |  |  |  | 8/8 | Low risk | Included |
| 1. **Ganhão C *et al*** | Y | Y | Y | Y | Y | UC | Y | Y |  |  |  | 7/8 | Low risk | Included |
| 1. **Nantale G *et al*** | UC | Y | Y | Y | Y | Y | Y | Y |  |  |  | 7/8 | Low risk | Included |

Key: Y=yes, N=no, UC=unclear, Q=Question
