# Supplementary material for: Magnitude of urban household food insecurity in East Africa: a systematic review and meta-analysis
Source: Public Health Nutr. 2021 Aug 16;25(4):994–1004. doi: 10.1017/S1368980021003529 (PMC9991803; doi:10.1017/S1368980021003529)
Supplement: Supplementary file 1 [file S1368980021003529sup.zip › S1368980021003529sup002.docx]

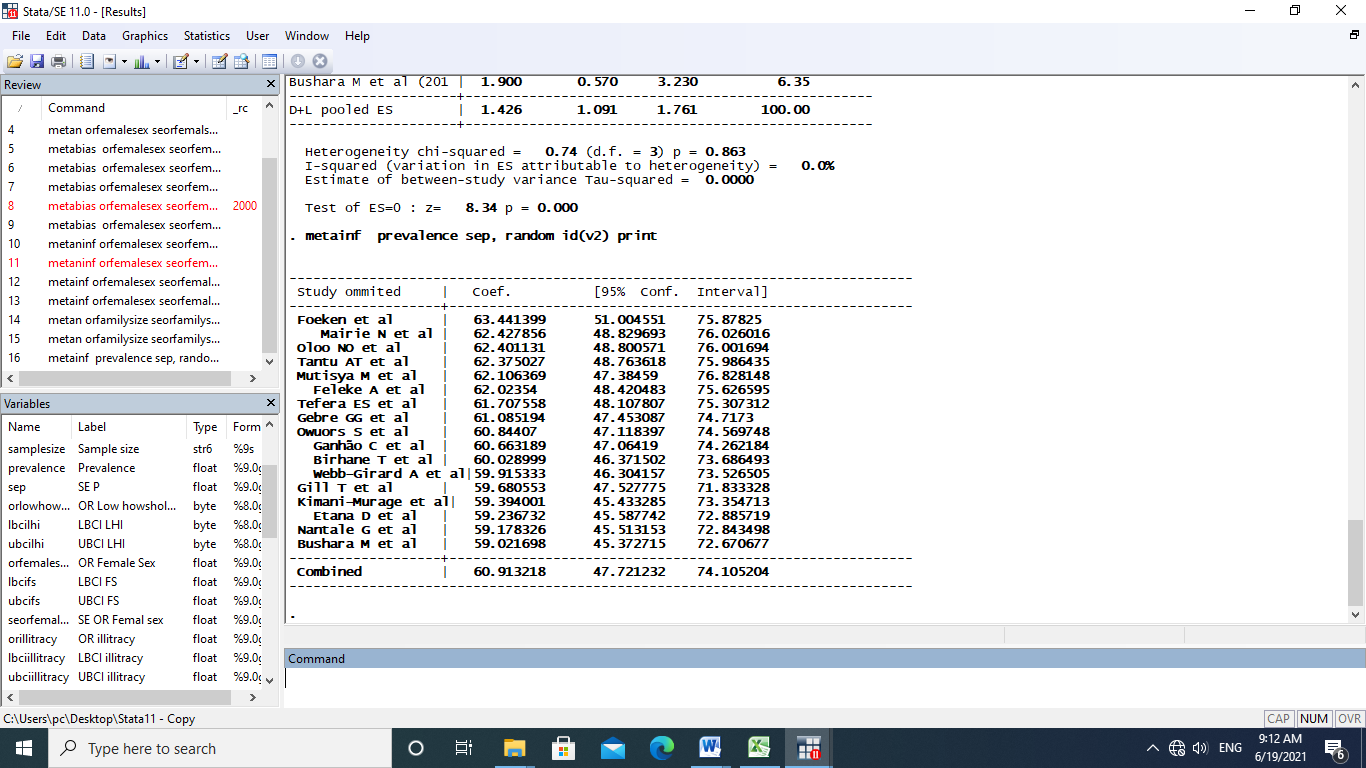


Supplementary Figure 1: Sensitivity analysis of the prevalence of urban household food insecurity in Eastern Africa.


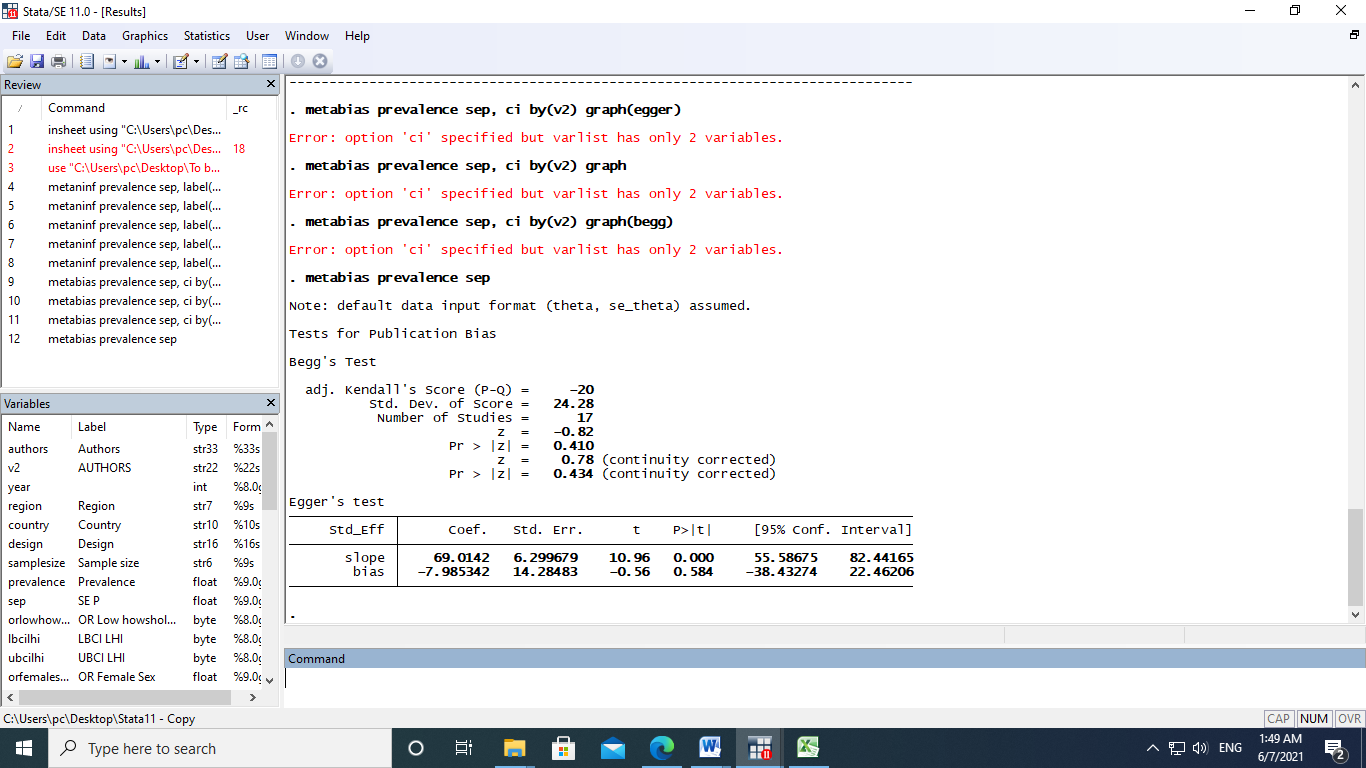
 Supplementary Figure 2: Eggers test of publication bias on the prevalence of urban household food insecurity in Eastern Africa.


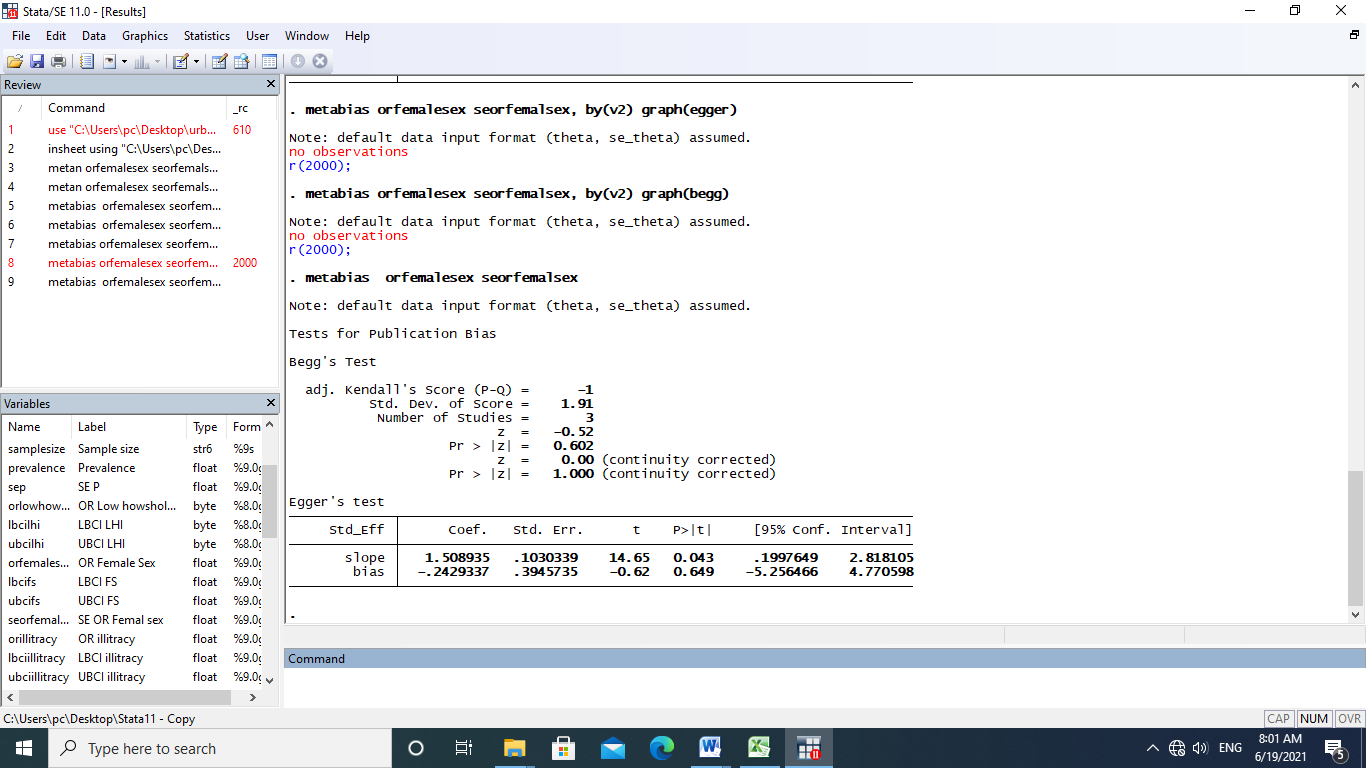


Supplementary Figure 3: Eggers test of publication bias on estimates of being female headed household as risk factors of urban household food insecurity in Eastern Africa


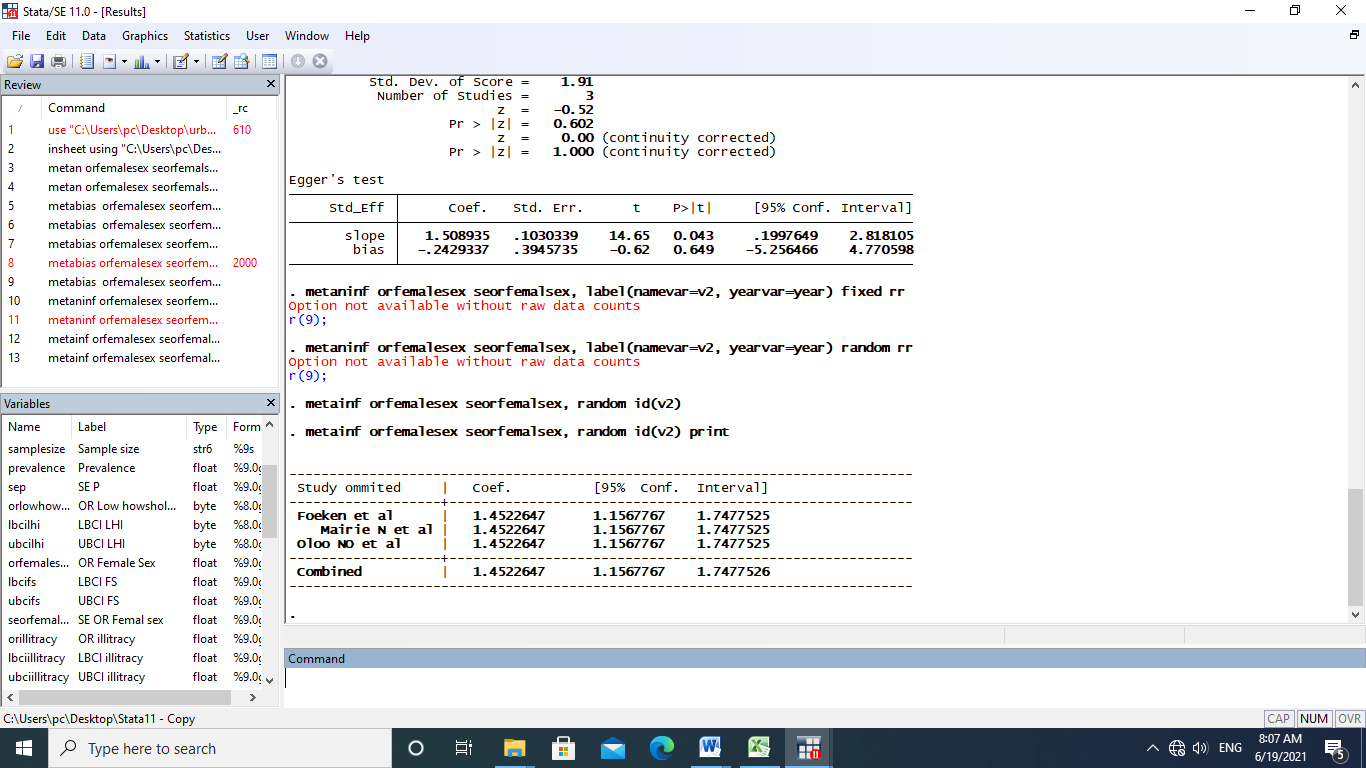


Supplementary Figure 4: Sensitivity analysis on estimates of being female headed household as risk factors of urban household food insecurity in Eastern Africa


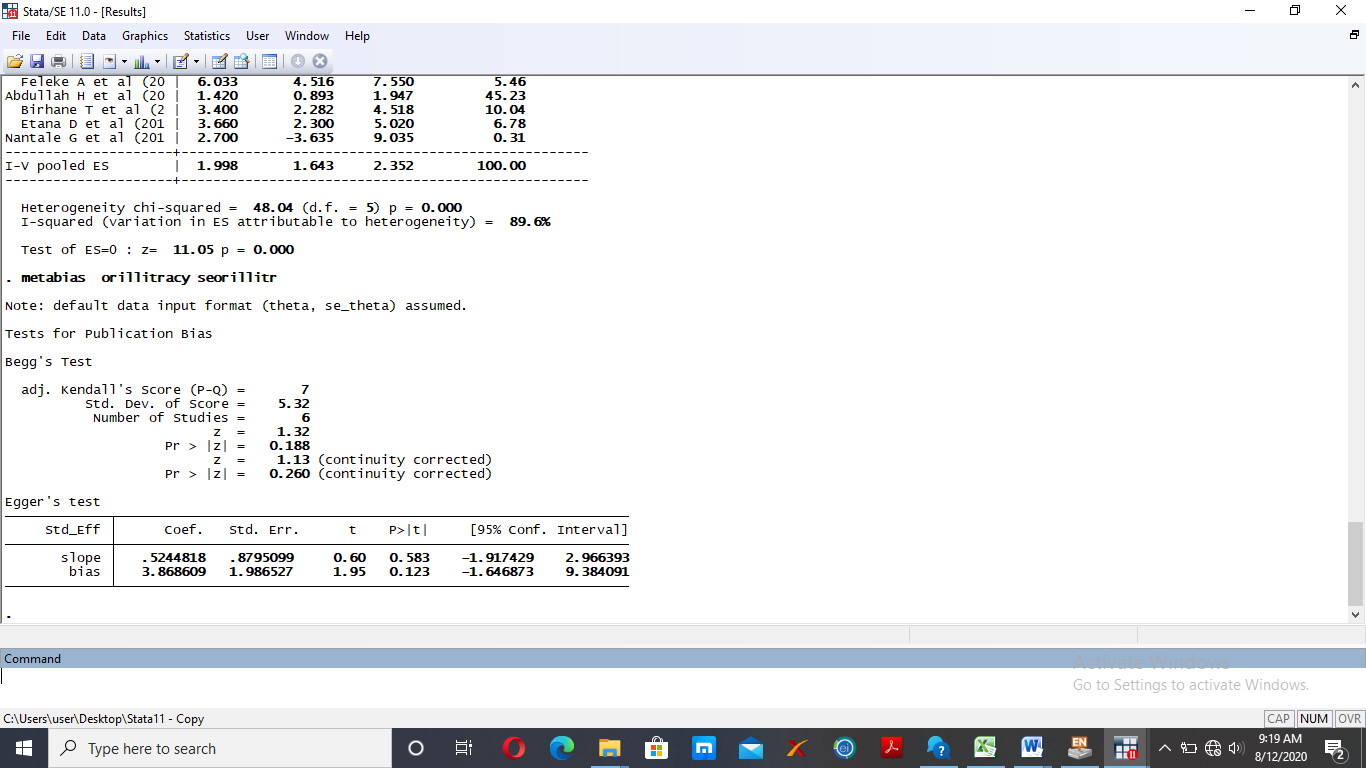


Supplementary Figure 5: Eggers test of publication bias on pooled estimate of illiteracy as risk factors of urban household food insecurity in Eastern Africa


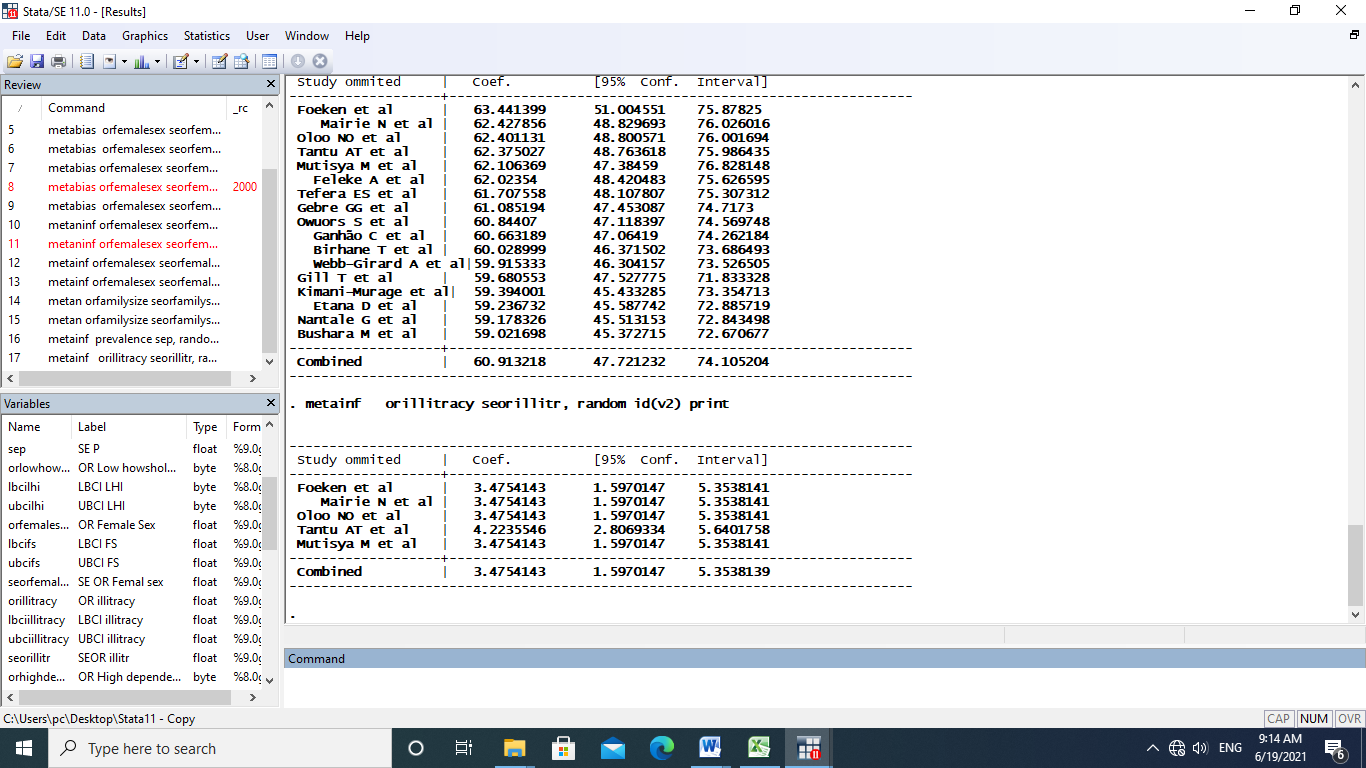


Supplementary Figure 6: Sensitivity analysis on pooled estimate of illiteracy as risk factors of urban household food insecurity in Eastern Africa


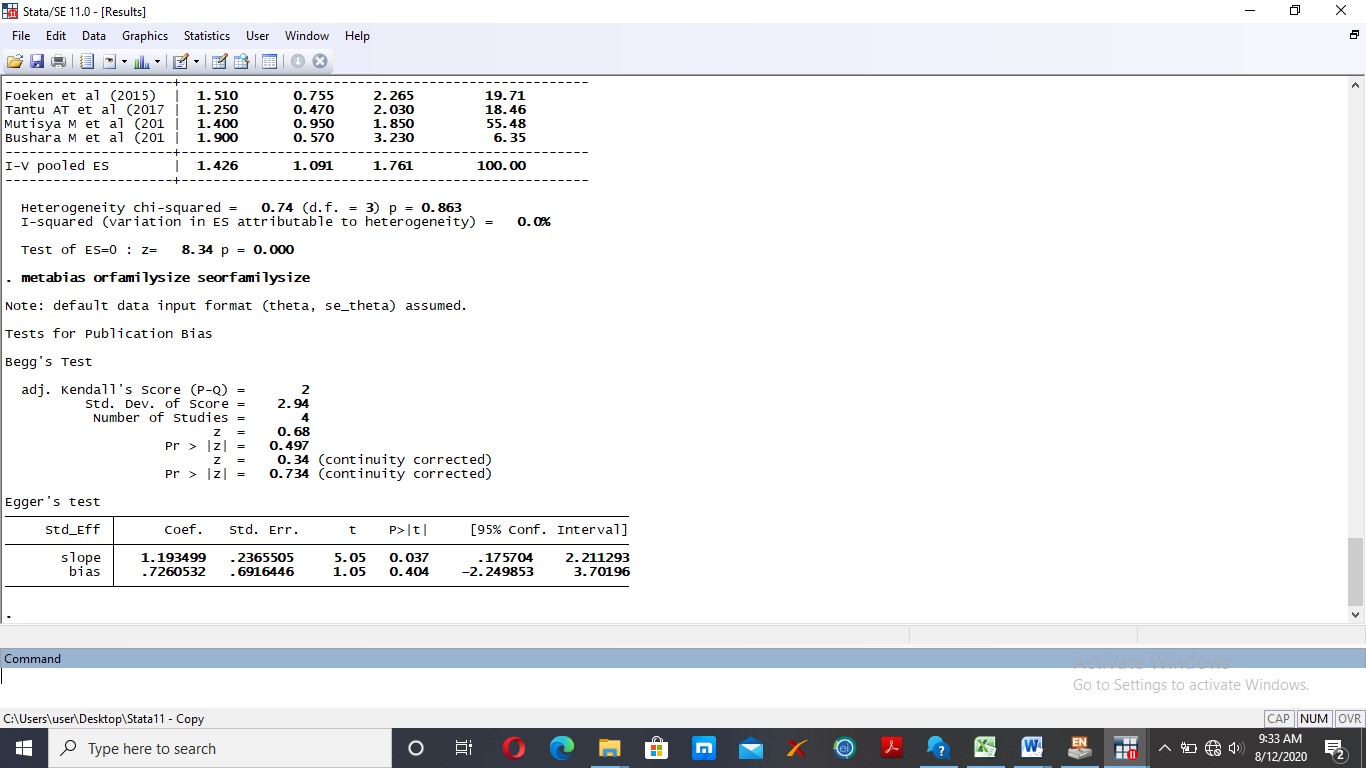


Supplementary Figure 7: Eggers test of publication bias on pooled estimate of increased family size as risk factors of urban household food insecurity in Eastern Africa


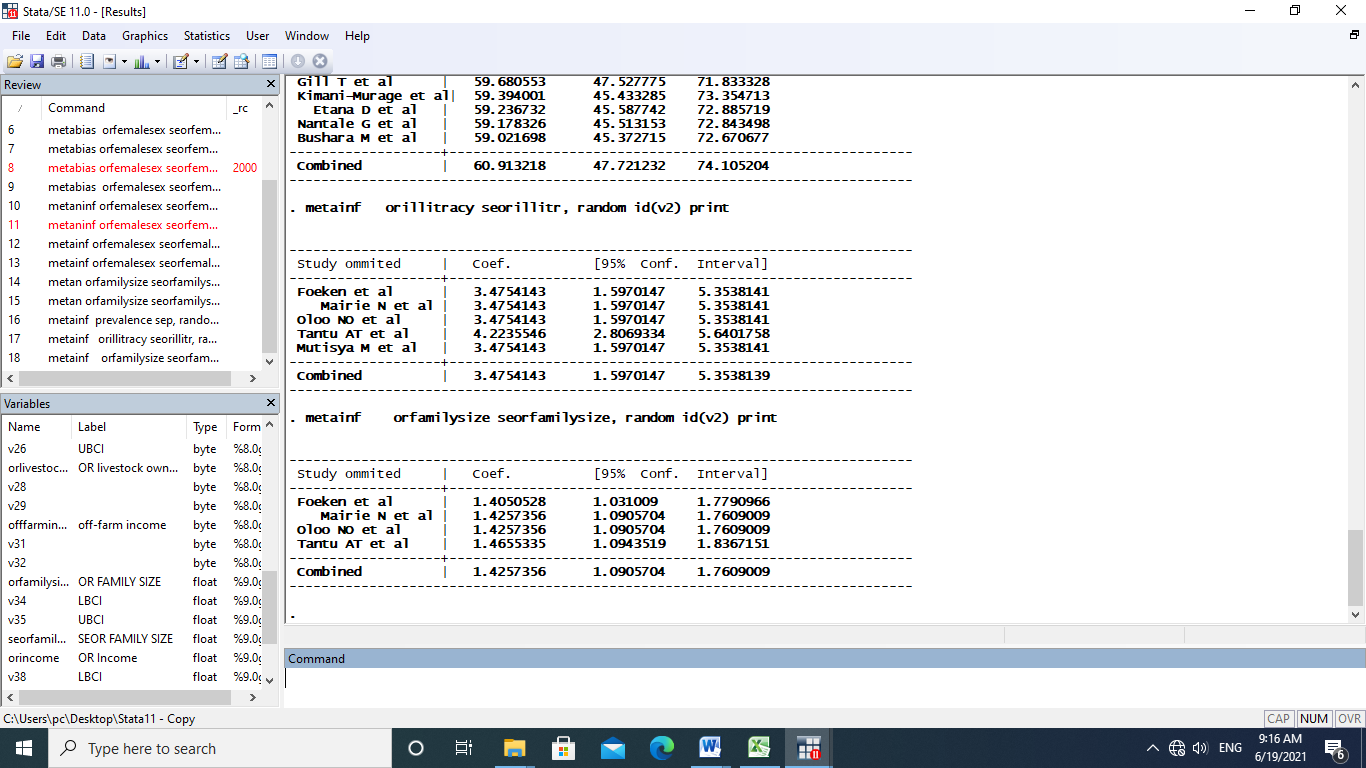


Supplementary Figure 8: Sensitivity analysis on pooled estimate of increased family size as risk factors of urban household food insecurity in Eastern Africa


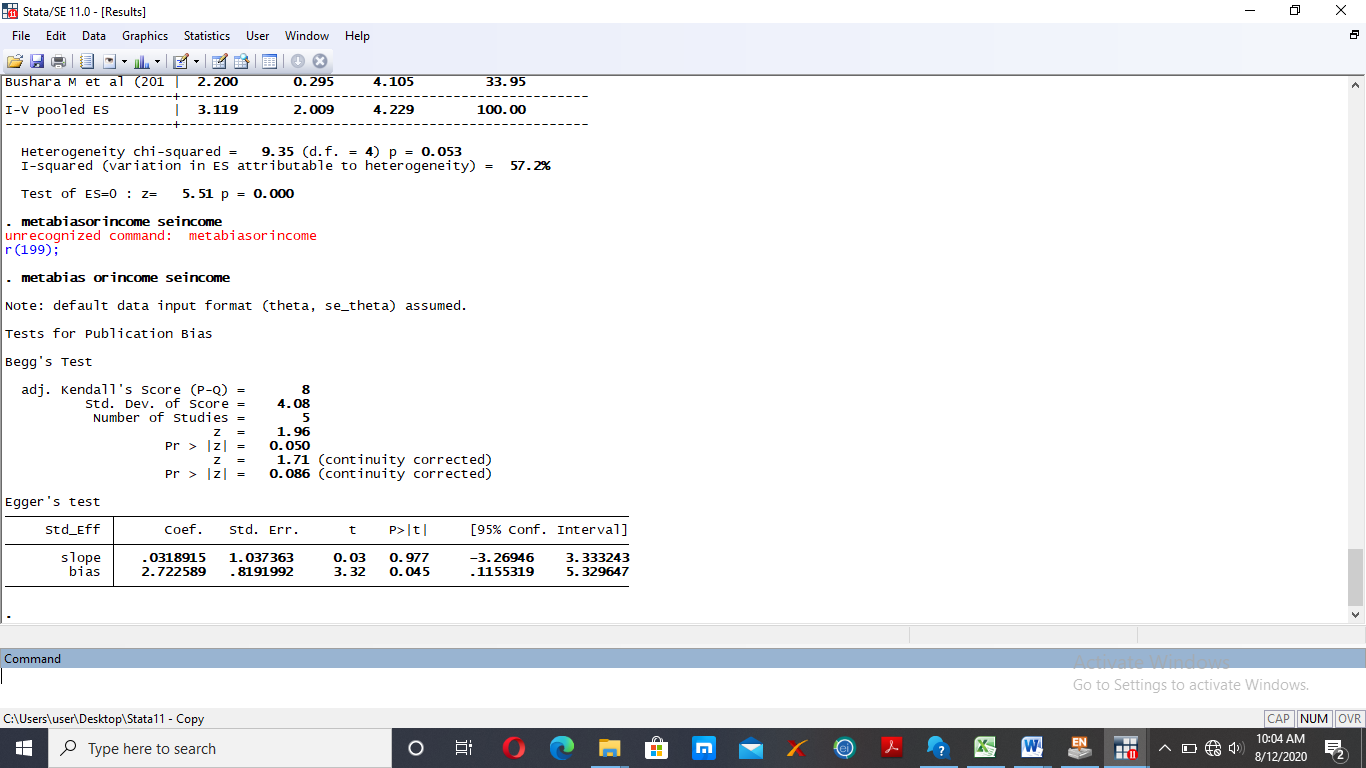


Supplementary Figure 9: Eggers test on the pooled estimate of low family income as risk factors of urban household food insecurity in Eastern Africa


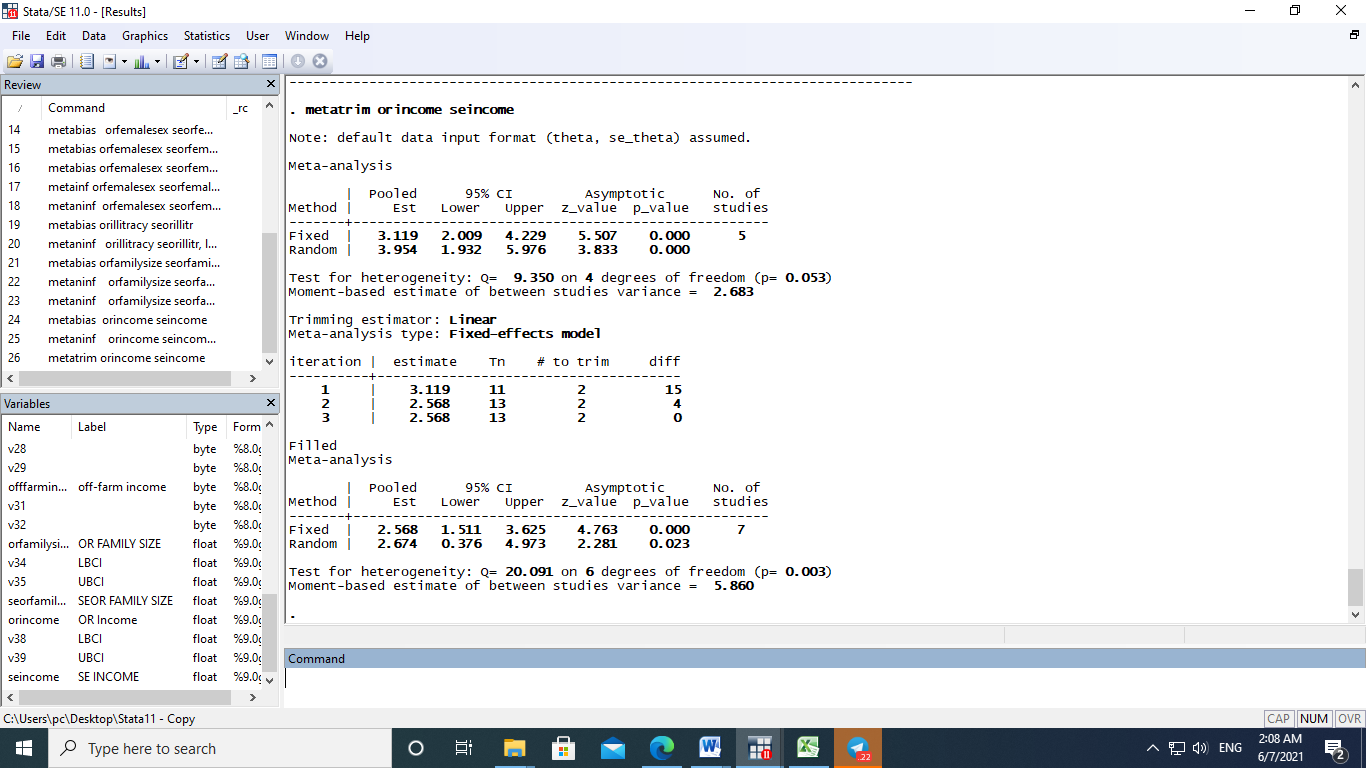


Supplementary Figure 10: Trim and fill analysis on the pooled estimate of low family income as risk factors of urban household food insecurity in Eastern Africa


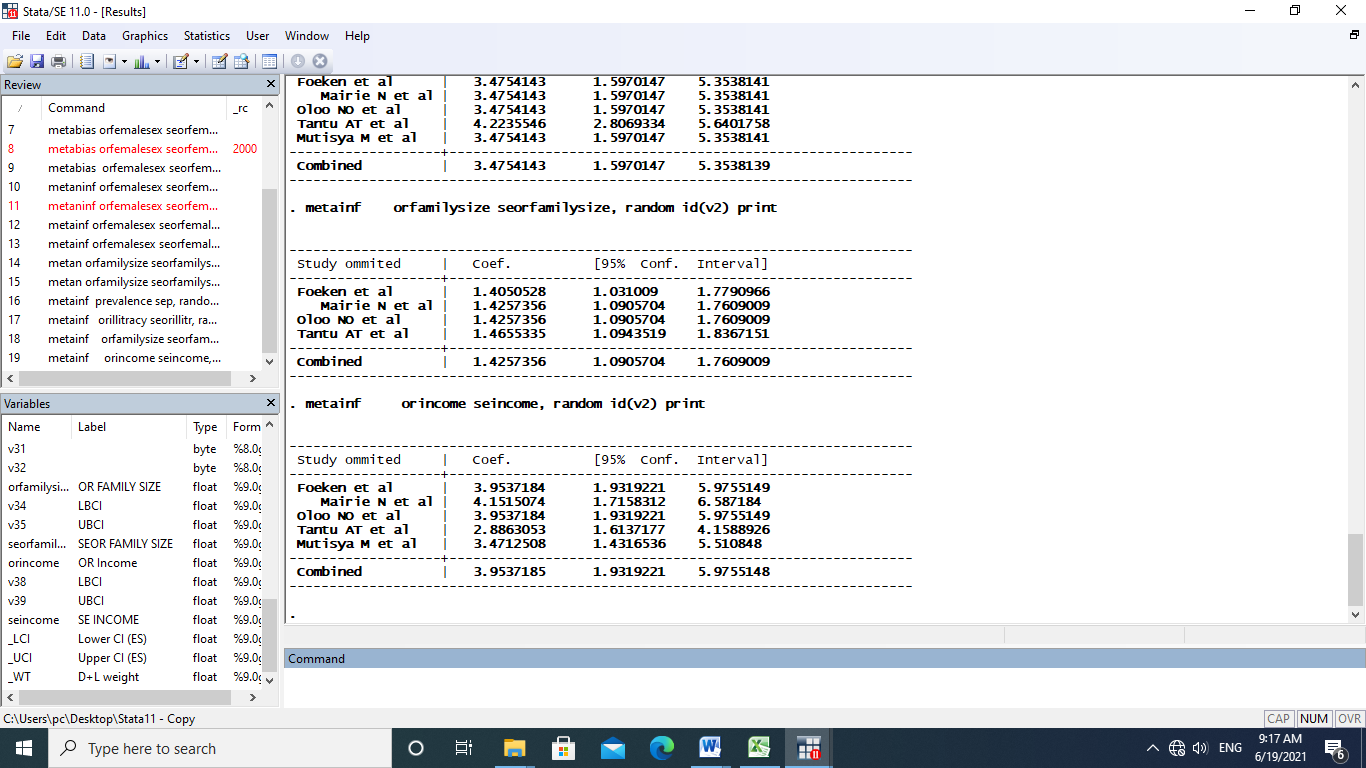


Supplementary Figure 11: Sensitivity analysis on the pooled estimate of low family income as risk factors of urban household food insecurity in Eastern Africa
